# Novel magnetically separable of Fe_3_O_4_/Ag_3_PO_4_@WO_3_ nanocomposites for enhanced photocatalytic and antibacterial activity against *Staphylococcus aureus* (*S. aureus*)

**DOI:** 10.1186/s12951-019-0485-z

**Published:** 2019-04-29

**Authors:** Hind Baballa Gasmalla, Xiaoquan Lu, Mahgoub Ibrahim Shinger, Lubin Ni, Aadil Nabi Chishti, Guowang Diao

**Affiliations:** 1 Key Laboratory of Environmental Materials & Environmental Engineering of Jiangsu Province, College of Chemistry and Chemistry Engineering, Yangzhou University, Yangzhou, 225002 China; 2 Forest Products and Industries Department, Faculty of Forestry, University of Khartoum, Khartoum, Sudan; 3 Key Laboratory of Bioelectrochemistry & Environmental Analysis of Gansu Province, College of Chemistry & Chemical Engineering, Northwest Normal University, Lanzhou, 730070 China; 4 Chemistry Department, Faculty of Science, International University of Africa, Khartoum, Sudan

**Keywords:** Fe_3_O_4_/Ag_3_PO_4_@WO_3_, Photocatalyst, Simulated light irradiation, Antibacterial activity

## Abstract

**Background:**

Iron oxide nanocomposites have received a great attention for their application in various fields like physics, medicine, biology, and material science etc., due to their unique properties, such as magnetism, electrical properties, small size, biocompatibility and low toxicity.

**Methods:**

Fe_3_O_4_/Ag_3_PO_4_@WO_3_ nanocomposites with different weight percent of Ag_3_PO_4_ were successfully prepared through fabricated Ag_3_PO_4_/Fe_3_O_4_ with WO_3_ via in situ fabrication method, electrospinning involved precursor solution preparation and spinning to enhance photocatalyst performance under simulated sunlight for the degradation of methylene blue (MB) and antibacterial activity against *Staphylococcus aureus* (*S. aureus*).

**Results:**

The photocatalytic degradation of methylene blue (MB) under simulated light irradiation indicated that the nanocomposite with 0.25 mg of Ag_3_PO_4_ has the best activity. An additional advantage of these photocatalysts is magnetic recoverability, using external magnetic field and photocatalytic stability of the nanocomposites was evaluated for three cycles. In addition, using different scavengers, holes (h^+^) and superoxide radical (O_2_^·−)^ radicals and hydroxide radical (^·^OH) were identified the main oxidative species in the degradation reaction of methylene blue.

**Conclusions:**

The results reveal that Fe_3_O_4_/Ag_3_PO_4_@WO_3_-0.25 nanocomposites have photocatalytic and antibacterial activity against *S. aureus.* The photocatalyst and mechanism based on the enhancement of electron transfer processes between Ag_3_PO_4_ and WO_3_ nanoparticles.

## Introduction

In recent years, research attention has been focused on processes that lead to an improved oxidative degradation of organic pollutants. Therefore, semiconductor photocatalysis technology has aroused scientists’ interest in environmental remediation. Although several semiconductors have proven to be ideal candidates for the treatment of water pollution, the efficient separation and recycling of this fine powdered photocatalyst is still a scientific problem when applied in practice, including separation process, selectivity, and dispersion [1, 2]. A photocatalyst with magnetic properties allow the use of the technique of magnetic separation, which is one of the most effective and simple methods for removing suspended solids from wastewater without need for further separation processes. The magnetic photocatalyst allows its use as a suspended material and providing the advantage to have a high surface area for reaction [3]. Several complexes such as Fe_2_O_3_, WO_3_, BiVO_4_, Bi_2_WO_6_ and Ag_3_PO_4_ [4–8] have been tested as visible light photocatalyst. Fe_3_O_4_ has been broadly applied as a significant ferromagnetic material for extensive application areas, including catalysis, recording materials, pigment, magnetocaloric refrigeration, and drug delivery carrier, because of its promising properties such as low cost, good hydrophilicity, and biocompatible properties [4]. Although Fe_3_O_4_ is not a suitable semiconductor for the photocatalyst process, it wasn’t expensive and possess high band gap energies as they willingly go through photocathodic corrosion [9, 10]. But the magnetic Ag_3_PO_4_/TiO_2_/Fe_3_O_4_ heterostructured nanocomposite was enhanced photocatalytic activity, cycling stability, and long-term durability in the photodegradation of acid orange 7 (AO7) under visible light. Moreover, the antibacterial activity toward *Escherichia coli* (*E. coli*) cells under visible-light irradiation [11].

Ag_3_PO_4_ has a relatively narrow band gap (2.36–2.43 eV) and is thus active under visible-light irradiation (λ < 530 nm). Therefore, as a highly efficient photocatalyst, Ag_3_PO_4_ could behave as a potential antimicrobial material and could have promise in various antimicrobial applications [12, 13]. Magnetically separable composite photocatalysts Fe_3_O_4_–Ag_3_PO_4_ [14], Ag_3_PO_4_/NiFe_2_O_4_ [15] and Fe_3_O_4_@LDH@Ag/Ag_3_PO_4_ [16] with high photocatalytic activity. Recently, magnetic and silver phosphate core–shell photocatalysts composed of a magnetic core and photocatalytic shell have attracted great interest Fe_3_O_4_@Ag_3_PO_4_/AgCl under simulated solar light [17] and Fe_3_O_4_@SiO_2_@Ag_3_PO_4_with excellent visible-light-responding photocatalytic activity [18]. WO_3_ has a narrow band gap (2.6–2.7 eV), is very hopeful visible light active photocatalyst. It has been used in the photocatalytic degradation of organic contaminants and in the photocatalytic development of O_2_ [19–21]. Micro and nanoscaled core–shell materials have attracted great interest because their essential properties can be conveniently adjusted by controlling the morphology and chemical composition of both core and shell [22–24]. Moreover, the interactions between the various ingredients of the core and shell can significantly improve the overall performance of the core–shell system and even produce beneficial synergistic effects [25, 26]. For example, metal–semiconductor as core–shell photocatalysts have been synthesized and shown to display superior photocatalytic efficiency, because a metallic core can accelerate charge separation and the large-surfaced nanostructures enhance light absorption [27]. However, the photocatalytic activity of WO_3_ is not reasonable because of its relatively low conduction band level. There are some reports on the synthesis of chestnut, such as Fe_3_O_4_/WO_3_ hierarchical core–shell structures that integrate conductive Fe_3_O_4_ microspheres and visible light active WO_3_ nanoplates as a magnetically recyclable visible light active photocatalyst [27]. In another study the fabrication of a magnetically recyclable Fe_3_O_4_/WO_3_ core–shell visible-light photocatalyst has been developed [28]. Nanosilver-decorated WO_3_ nanofibers incorporating paramagnetic CoFe_2_O_4_ nanoparticles were fabricated for the first time as model solar light-active photocatalyst with potent antibacterial property and recoverability. Formation of semiconductor composites is an effective way to enhance the photo-induced charges separation efficiency and the photocatalytic performance, which has been extensively studied for the last decades [20].

Magnetic photocatalysts are recognized as low cost, efficient, and robust techniques desirable to remove dyes from contaminated water before their discharge and to produce clean water on a large scale. In principle, the rapid separation of photogenerated electrons and holes can be achieved by forming heterojunctions within the semiconductors. Further, multiscale structuring can beneficially increase the light scattering and absorption in such heterojunctions, thus increasing the light absorption range [29]. Hence, such a strategy can be successfully used for the fabrication of high-efficiency semiconductor composites.

In the present work, through in situ ion exchange method, we fabricated Ag_3_PO_4_/Fe_3_O_4_ with WO_3_ for the first time as model simulated light-active photocatalyst for the degradation of methylene blue (MB). The antibacterial activity of Ag_3_PO_4_/Fe_3_O_4_@WO_3_ composite toward *Staphylococcus aureus* (*S. aureus*) was studied. The as synthesized Ag_3_PO_4_/Fe_3_O_4_@WO_3_ composite exhibited high antibacterial performance than the other prepared composites. The effect of the presence of the reactive species on the photocatalytic activity was studied, and the possible photocatalytic mechanism of Fe_3_O_4_/Ag_3_PO_4_@WO_3_ and photodegradation pathway of MB was suggested.

## Experimental section

### Materials

All materials have analytical purity and used as received without further purification. All of the solutions were prepared using deionized water (18.2 MΩ). FeSO_4_·7H_2_O, FeCl_3_·6H_2_O, NH_4_OH and Disodium ethylenediamine tetraacetate (Na_2_-EDTA) solution were obtained from Yantai both chemical Co., Ltd, AgNO_3_ (99.8%), MDF and *p*-benzoquinone (BZQ) were obtained from Tianjin Chemical reagent technology Co., Ltd. Disodium hydrogen phosphate dodecahydrate (Na_2_HPO_4_·12H_2_O) and H_2_WO_4_ (AR, 99.95%) was bought from Aladdin reagent Co., Ltd., Ethanol (AR, 99.7%), was purchased from Tianjin Baishi Chemical Co., Ltd. PVP, MB and tert-butanol obtained from Tianjin city Tianxin chemicals development center, Nutrient Broth powder was obtained from Hanzhou Microbiology Co., Ltd and agar powder was obtained from Beijing life science and technology. Co., Ltd.

### Preparation Fe_3_O_4_ nanoparticles

The functionalized Fe_3_O_4_ was prepared by the modified co-precipitation method. Typically, 2.7 g FeSO_4_·7H_2_O and 5.4 g FeCl_3_·6H_2_O were dissolved in 50 ml deionized water separately, and mixed together in water bath maintained at 80 °C. Then 2 ml of oleic acid (OA) was added into the FeSO_4_/FeCl_3_ mixture, and sonicated for 20 min under 60 °C, then 15 ml of ammonium hydroxide was added drop by drop with stirring (600 revolutions/min) under nitrogen atmosphere. After 0.5 h, 2 ml of OA was added again drop-by-drop to the mixture with stirring and reacted for 1 h at 80 °C under nitrogen gas. After that, the precipitate was isolated from the solvent by a permanent magnet. Then washed with water and ethanol in sequence, followed by drying at 50 °C under vacuum for 24 h. Non-functionalized Fe_3_O_4_ was prepared following the same method without the addition of OA, and labeled as Fe_3_O_4_-non.

### Preparation of Fe_3_O_4_/Ag_3_PO_4_

For the preparation of Fe_3_O_4_/Ag_3_PO_4_, room temperature in situ anion-exchange method was used. Typically, 0.5 g of the as prepared Fe_3_O_4_ was dispersed into 50 ml distilled water and sonicated for 1 h to give black aqueous suspension and then 10 ml of three different amount of AgNO_3_ (0.25 g, 0.50 g and 0.75 g) was added into the suspension solution and stirred for 0.5 h. Subsequently, 10 ml of aqueous solution with different content of Na_2_HPO_4_ (0.13 g, 0.26 g, 0.39 g) was added drop-by-drop with continues stirring for more 0.5 h. Finally, the precipitates were separated by a permanent magnet and washed with distilled water several time and dried under vacuum for 24 h. The obtained composites were labeled as Fe_3_O_4_/Ag_3_PO_4_-0.25, Fe_3_O_4_/Ag_3_PO_4_-0.5 and Fe_3_O_4_/Ag_3_PO_4_-0.75. Fe_3_O_4_/Ag_3_PO_4_-non was prepared using Fe_3_O_4_-non and (0.25 g/10 ml) AgNO_3_ and (0.13 g/10 ml) Na_2_HPO_4_.

### Preparation of tungsten oxide WO_3_

Fabrication of WO_3_ square like nanoplates by electrospinning involved precursor solution preparation and spinning. Combination of a polymer solution and a tungsten oxide precursor solution resulted in precursor gel for electrospinning. The polymer solution could be prepared by dissolving a selected polymer in a solvent while the tungsten oxide precursor solution by dissolving the salt in a suitable solvent. The following protocol is given as a specific example; A PVP solution was prepared by adding 0.7 g of polyvinylpyrrolidone into 7 ml of ethanol under magnetic stirring. In a separate beaker, 0.2 g of tungstic acid was added to 2 ml of DMF. Then, the two solutions were mixed followed by magnetic stirring at room temperature for 15 min. Then the precipitates were separated by a permanent magnet and washed with distilled water several time and dried under vacuum for 24 h.

### Preparation of Fe_3_O_4_/Ag_3_PO_4_@WO_3_

The fabrication of magnetic with silver phosphate involved WO_3_ precursor solution preparation and electrospinning was chosen as a facile and an effective approach to fabricate plates with diameters at nanometer length scale. The precursor solutions were derived from mixtures Fe_3_O_4_/Ag_3_PO_4_ nanoparticles with different concentration and tungsten oxide precursor. A typical method is as follows. A PVP solution was prepared by adding 0.7 g of polyvinylpyrrolidone into 7 ml of ethanol under magnetic stirring. In a separate beaker, 0.2 g of tungsten acid was added to 2 ml of DMF. Then, the two solutions were mixed followed by magnetic stirring at room temperature for 15 min. To the mixture above, determined about 0.1 mg of different amounts of Fe_3_O_4_@Ag_3_PO_4_ nanoparticles were added. The final mixture was then stirred by a magnetic stirrer at room temperature for 15 min, followed by sonication for 10 min. Then the precipitates were separated by a permanent magnet and washed with distilled water several time and dried under vacuum for 24 h. The obtained composites were labeled as Fe_3_O_4_/Ag_3_PO_4_@WO_3_-0.25, Fe_3_O_4_/Ag_3_PO_4_@WO_3_-0.05 and Fe_3_O_4_/Ag_3_PO_4_@WO_3_-0.75. Non-functionalized Fe_3_O_4_/Ag_3_PO_4_@WO_3_ was synthesized by the same method using Fe_3_O_4_/Ag_3_PO_4_-non instead of Fe_3_O_4_/Ag_3_PO_4_, and labeled as Fe_3_O_4_/Ag_3_PO_4_@WO_3_-non.

### Characterization

The morphology and the composition were characterized; Transmission Electron Microscopy (TEM, FEI TECNAI-G2 operating at 300 kV) and Field-emission Scanning Electron Microscope (FESEM, JEOL, JSM-7001F) were used to determine the morphology and size of the magnetite particles, The structures of composites were characterized by X-ray diffractometer (XRD) using a Brucker 8 Advanced, Germany with a Cu Kα (λ = 1.5406˚ A) source in the 2θ range of 10 ° to 80 ° at room temperature, A vibration sample magnetometer VSM (Model EV9System) was employed for magnetic properties of the samples at room temperature, The Fourier Transform Infrared (FTIR) spectra of the samples were recorded on a Bruker Vertex 70 FT-IR spectrophotometer using the KBr method, The UV–visible diffuse reflectance spectroscopy (DRS) spectra of photocatalyst powder was obtained for the dry-pressed disk samples using scan Shimazu, Japan UV-2450 spectrometer equipped with the integrated sphere accessory for diffuse reflectance spectra, using BaSO_4_ as the reflectance sample, UV–vis absorption spectra of the as-prepared samples were obtained using a Hitachi U-4500 spectrophotometer (Hitachi High-Technology Corporation). The electron transfer properties of the synthesized composites were studied using electrochemical impedance spectrometer (EIS) VMP2 multi-potentiostat with ZsimpWin program (Princeton Applied Research, USA) and it’s frequencies swept from 10 kHz to 100 mHz.

### Evaluation of the antibacterial activity

#### Preparation of photocatalyst film

In general, suspensions of the as-prepared Fe_3_O_4_/Ag_3_PO_4_@WO_3_ composites (0.001 mg/1 ml) were dripped onto filter paper (Φ 6 mm), and then placed onto the bottom of a Petri dish (nutrient agar plate) for antibacterial experiments. For comparison, the same film was prepared using Fe_3_O_4_, Ag_3_PO_4_ or WO_3_.

#### Antibacterial tests

The antibacterial activity was evaluated using *S. aureus* and *E. coli* as representative microorganisms. Before the antimicrobial experiments, all glass wares were sterilized by autoclaving at 120 °C for 30 min. To measure antibacterial activity, 100 μl of the bacterial suspension was serially diluted with sterile water to make sure the final colony count is not more than 10^−7^ colony-forming units per milliliter (CFU ml^−1^). Then 100 μl aliquots were spread onto nutrient agar plates that were prepared already and incubated at 37 °C for 24 h. The experiment was further repeated. For each antibacterial experiment, the prepared photocatalyst film was placed onto the bottom of a Petri dish (onto an agar plate) seeded with 100 μl of *S. aureus* or *E. coli*. After 24 h of incubation at 37 °C, the diameters of the inhibition zones were measured.

#### Photocatalytic activity

The photocatalytic evaluation study was carried out at room temperature. Typically, MB solution (50 ml, 5 mg l^−1^) and 15 mg of the catalyst were placed in a sealed glass. The suspension was ultrasonicated in the dark for 10 min before irradiation to reach an adsorption/desorption equilibrium between MB and the surface of the catalyst under room temperature. Then the mixture was illuminated with 350 Xe lamp as a light source system equipped with a UV cutoff filter (λ > 420 nm). The distance between the light source and the reaction containers was fixed at 10 cm. After simulated light irritation, 3 ml of the mixture was taken out at a regular time interval of 1 min. Then centrifuged to remove the photocatalyst and the concentration of MB was measured with UV–Vis spectrophotometer at 664 nm.

#### Analysis of hydroxyl radical (^·^OH)

Terephthalic acid photoluminescence probe technique was used in the detection of ^·^OH. Terephthalic acid readily reacted with OH to produce highly fluorescent product, 2-hydroxy-terephthalic acid. The method relied on the PL signal at 426 nm of 2-hydroxyterephthalic acid. The PL intensity of 2-hydroxyterephthalic acid was proportional to the amount of ^·^OH formed. Experimental procedures were reported in early reports [30, 31], a basic terephthalic acid solution was added to the reactor and the concentration of terephthalic acid was set at 5 × 10^−4^ M in 2 × 10^−3^ M NaOH solution. The solution was irradiated for intervals time 10 min using ultraviolet light 365 for 50 min under magnetic stirring. The PL spectra of generated 2-hydroxyterephthalic acid were measured on a Hitachi F-4500 fluorescence spectrophotometer. The reaction solution was used to measure the increase of the PL intensity at 426 nm excited by 365 nm ultraviolet light.

## Result and discussion

### Characterization

#### Transmission electron microscopy

**Fig. 1:**
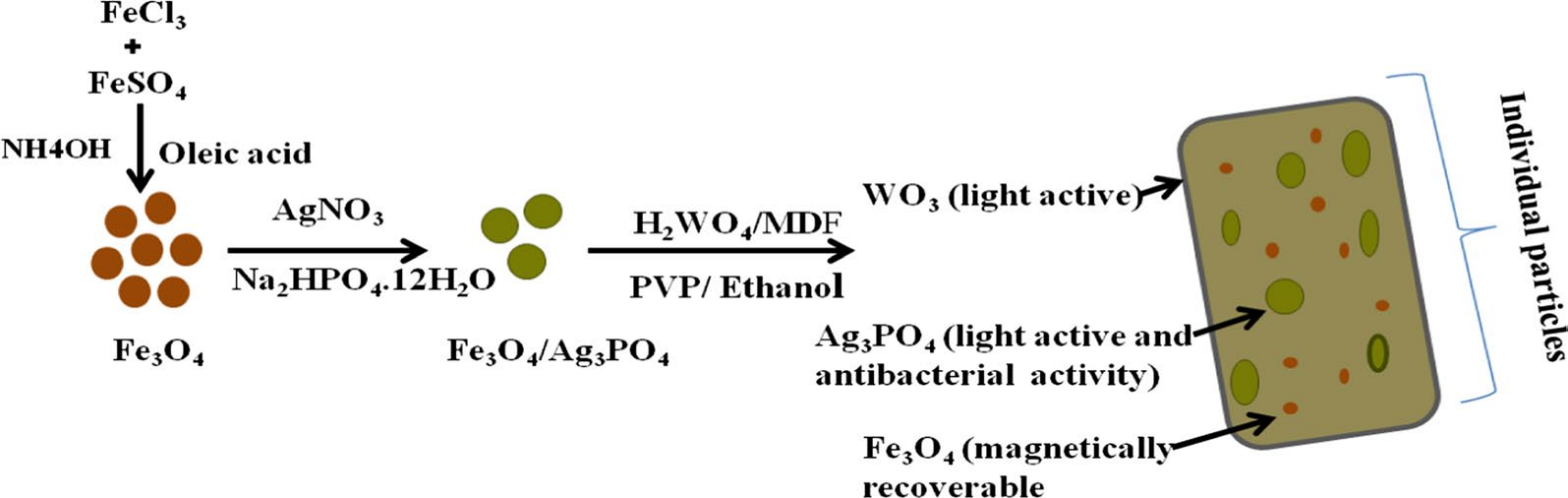
Scheme diagram for the preparation of Fe_3_O_4_/Ag_3_PO_4_@WO_3_ composite

This synthesis procedure of Fe_3_O_4_/Ag_3_PO_4_@WO_3_ nanocomposites is presented Schematic diagram in Fig. 1. A Fe_3_O_4_ nanoparticle was first synthesized by co-precipitation method and fabrication of magnetic with silver phosphate involved WO_3_ precursor solution preparation and electrospinning method.

**Fig. 2:**
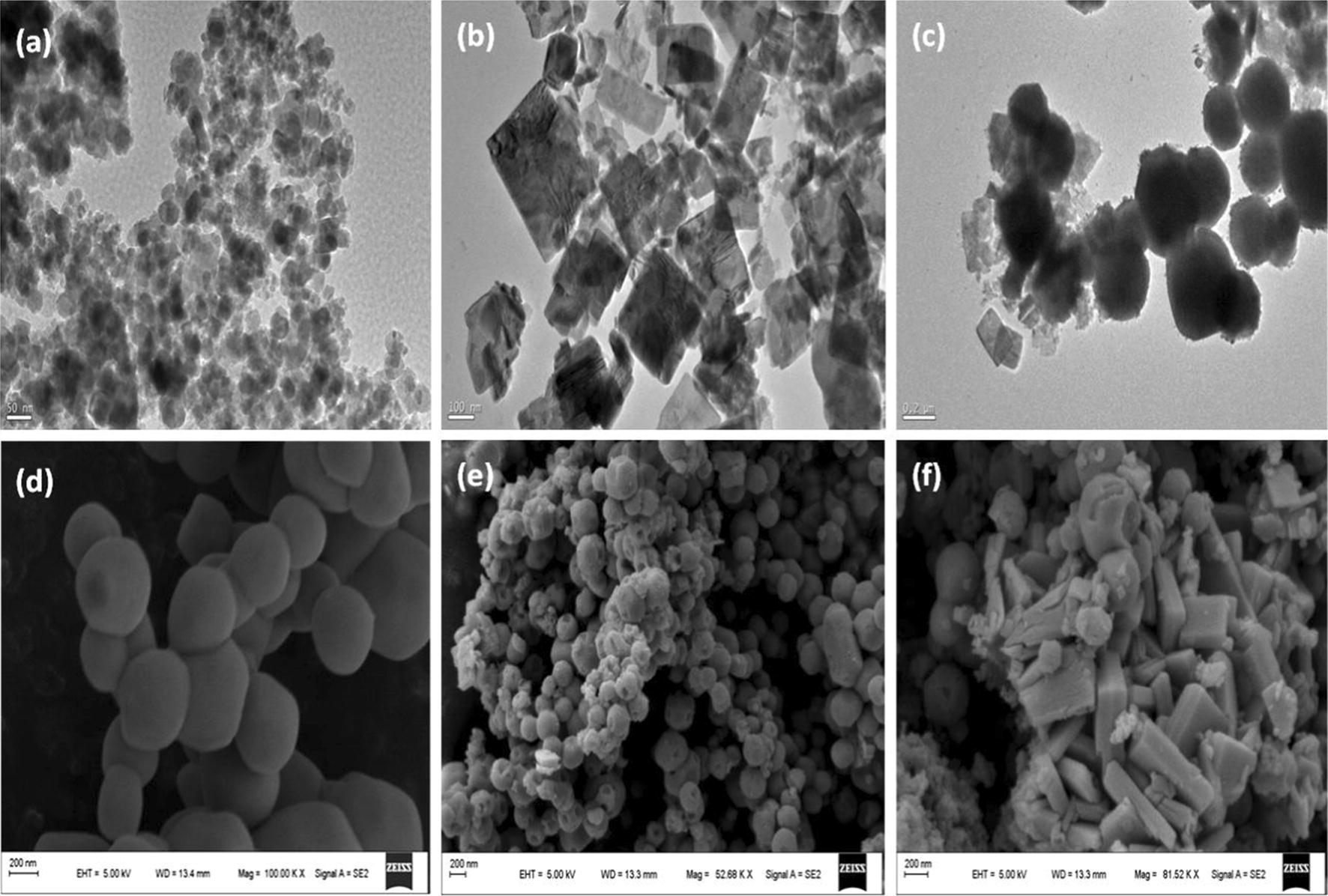
TEM image of **a** Fe_3_O_4_, **b** WO_3_ and **c** Fe_3_O_4_/Ag_3_PO_4_@WO_3_-0.25 and the SEM image of **d** Ag_3_PO_4_, **e** Ag_3_PO_4/_Fe_3_O_4_ and **f** Fe_3_O_4_/Ag_3_PO_4_@WO_3_-0.25

Figure 2a–c presents typical TEM images of the bare Fe_3_O_4_, Ag_3_PO_4_ and WO_3_, Fe_3_O_4_/Ag_3_PO_4_@WO_3_ composites, respectively. As seen in Fig. 2c, the as prepared Fe_3_O_4_/Ag_3_PO_4_ well cabled with the WO_3_ nanoplates, indicating the Fe_3_O_4_/Ag_3_PO_4_@WO_3_ composite has been successfully fabricated spherical shape with wide size distribution, and the aggregation of the particles is dense and the size range of bare Fe_3_O_4_ nanoparticles is 29 nm Fig. 2d–f shows the SEM images of the Ag_3_PO_4_, Fe_3_O_4_/Ag_3_PO_4_, and Fe_3_O_4_/Ag_3_PO_4_@WO_3_ composites, respectively. As been seen in (Fig. 2e), the Fe_3_O_4_ nanoparticles obviously decorated the Ag_3_PO_4_. While the SEM image of the Fe_3_O_4_/Ag_3_PO_4_@WO_3_ shows the presence of Fe_3_O_4_, Ag_3_PO_4_ and WO_3_. Which further confirm the fabrication of Fe_3_O_4_/Ag_3_PO_4_@WO_3_.

#### X-Ray energy-dispersive spectroscopy analysis

**Fig. 3:**
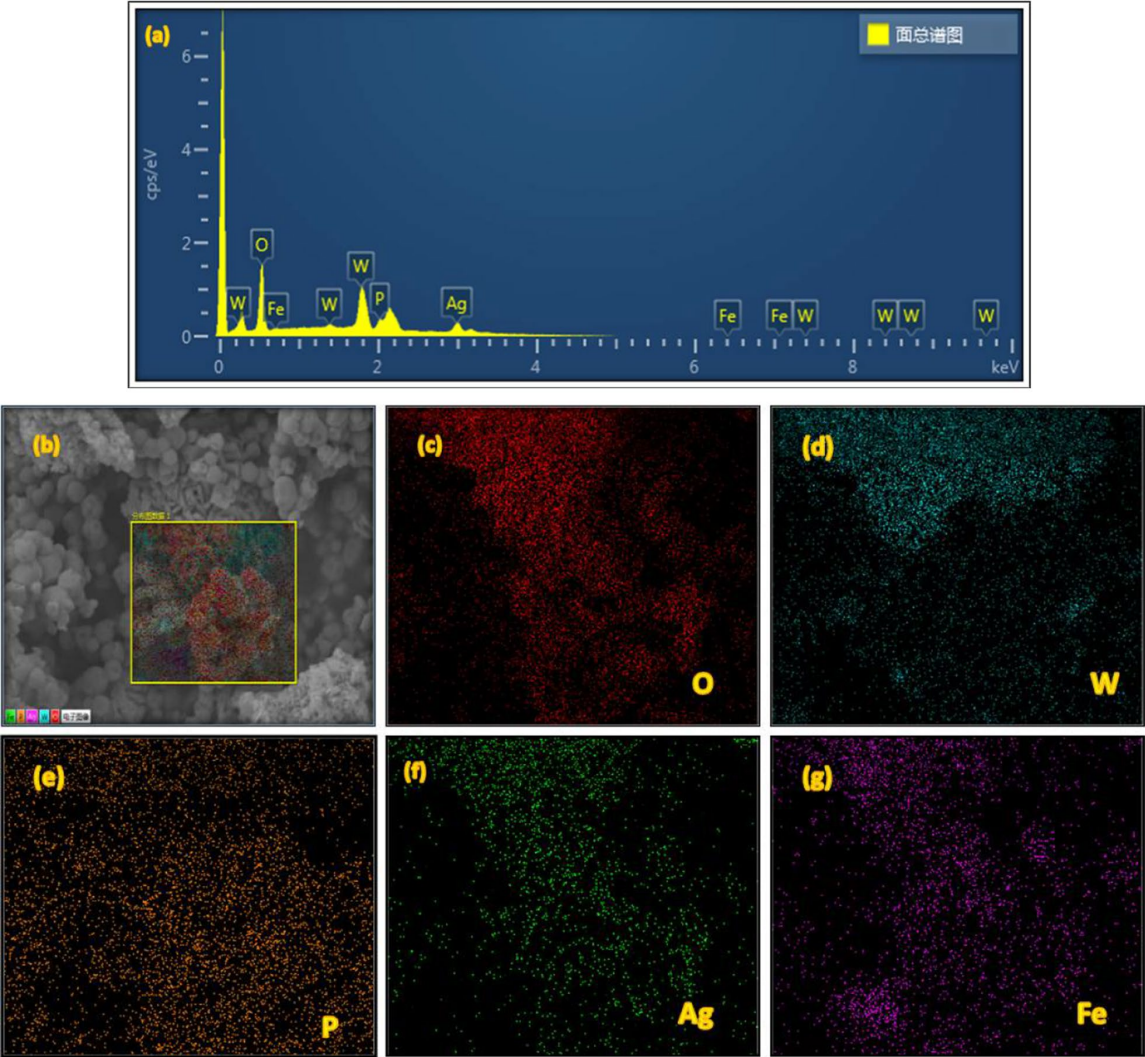
**a** EDS spectra for the F_3_O_4_/Ag_3_PO_4_@WO_3_, **b**–**g** EDS mapping for the F_3_O_4_/Ag_3_PO_4_@WO_3_ nanocomposite

The purity of the samples was investigated using EDS analysis and the results are shown in Fig. 3a. As can be seen the peaks in the spectra of the Fe_3_O_4_/Ag_3_PO_4_@WO_3_-0.25 composite are described to O, W, P, Fe, and Ag elements. Moreover, the EDS elemental mapping further elucidated the composition of the Fe_3_O_4_/Ag_3_PO_4_@WO_3_-0.25 nanocomposite. As can be seen in Fig. 3b–g, the elemental mapping images of O, W, P, Fe and Ag have similar shape and location, indicating the definite existence of Fe_3_O_4_, Ag_3_PO_4_, and WO_3_.

#### X-ray diffraction

**Fig. 4:**
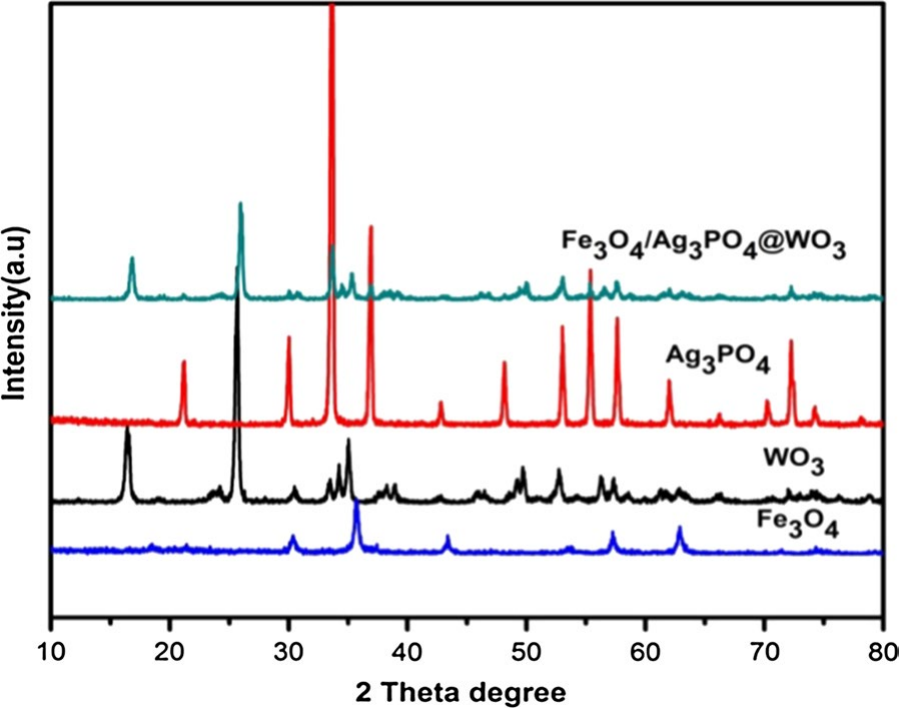
XRD patterns of Fe_3_O_4_, Ag_3_PO_4_, WO_3_ and F_3_O_4_/Ag_3_PO_4_@WO_3_

The XRD analysis was used to investigate the different crystalline structures of the synthesized composites (Fig. 4). The Fe_3_O_4_ shows six characteristic peaks at 2θ = 30.1, 35.3, 43.6, 53.6, 57.3 and 62.8, indexed to (220), (311), (400), (422), (511) and (440) facets, respectively, which can be well indexed to the standard data (JCPDS File, no: 19-0629) [32]. The XRD pattern of Ag_3_PO_4_ shows diffraction peaks at 2θ = 20.848, 29.648, 33.248, 36.518, 42.428, 47.718, 52.608, 54.928, 57.188, 61.548, 65.728, 69.788, 71.768, and 73.728 corresponded to the planes (110), (200), (210), (211), (220), (310), (222), (320), (321), (400), (330), (420), (421) and (332) of Ag_3_PO_4_, respectively, which can be indexed to the standard XRD data of the cubic-phase Ag_3_PO_4_ crystal (JCPDS File, no: 06-0505, 74-1876) [33, 34]. The square-like WO_3_ nanoplates indicate that the as-synthesized WO_3_ crystal structure with obvious diffraction peaks at 2θ value of 16.5° (020), 19.2.8° (011), 23.8° (120), 25.8° (111), 35.5° (131), and 78.6° (313) which are similar to the pattern of the reference WO_3_ crystals (JCPDS File no: 84-886) [35]. The XRD pattern of Fe_3_O_4_/Ag_3_PO_4_@WO_3_ composite displayed all the characteristic peaks of Fe_3_O_4_, Ag_3_PO_4_ and WO_3_, and no other peaks were detected. This further confirms the fabrication of the synthesized composite. The particle size play important role on the photocatalytic performance of the semiconductors, the less particle sizes the higher catalytic activity. To calculate the particle size of the synthesized composites, Scherrer’s Eq. 1 [36] was used.

1 $${\text{D}} = {\text{K}}\uplambda/\upbeta{ \cos }\theta$$

where *β* is full width at half-maximum, *D* is crystallite size, *λ* is X-ray wavelength, *θ* is Bragg’s diffraction angle.

The average sizes of the WO_3_ were estimated to be 450, 411 and 383 corresponding to the patterns of Fe_3_O_4_/Ag_3_PO_4_ and Fe_3_O_4_/Ag_3_PO_4_@WO_3_, respectively. From the calculations, the crystalline size of Fe_3_O_4_/Ag_3_PO_4_ markedly decreased after the synthesis of Fe_3_O_4_/Ag_3_PO_4_@WO_3_ composite. Which it’s beneficial enhance the photocatalytic activity.

#### Magnetic properties

**Fig. 5:**
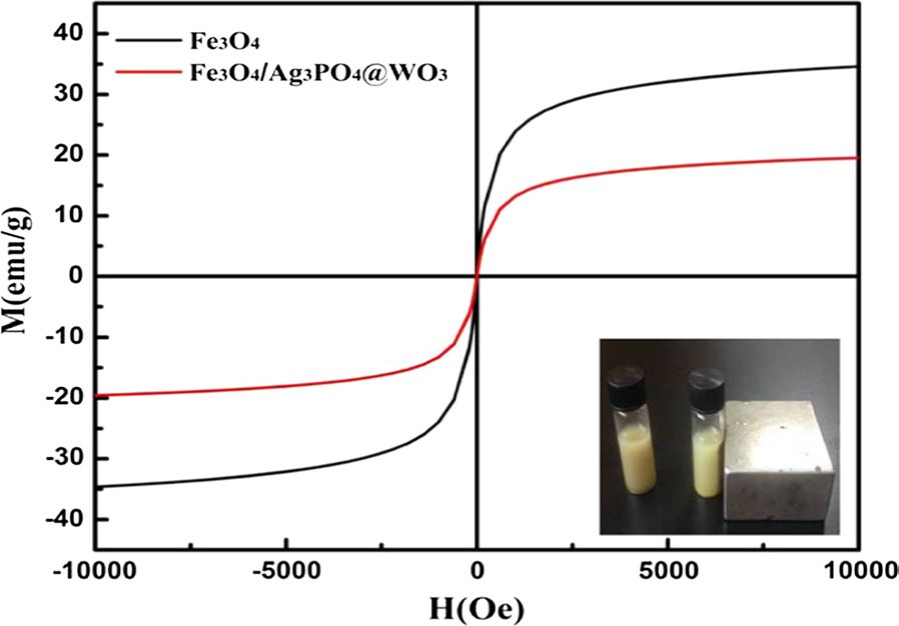
Magnetic hysteresis loops of Fe_3_O_4_ and Fe_3_O_4_/Ag_3_PO_4_ @WO_3_ composites catalyst. Inset indicate attraction of magnetic nanoparticles to magnet

The magnetic properties have been quantified by using a superconducting quantum interference device a vibrating sample magnetometer (VSM Model EV9System) at room temperature. Figure 5, displayed the hysteresis loops at 300 K of the as prepared Fe_3_O_4_ microspheres and Fe_3_O_4_/Ag_3_PO_4_@WO_3_-0.25 composites catalysts. The saturation magnetization (Ms), remanence (Mr), coercivity (Hc) and loop Squarenses ratio (Mr/Ms) of samples is shown in (Tables 1 and 2) and the magnetization curve with a hysteresis loop displays ferromagnetic behavior with a coercivity of about 2.20 and 1.87Oe of Fe_3_O_4_ and Fe_3_O_4_/Ag_3_PO_4_@WO_3_ respectively. The saturation magnetization of the Fe_3_O_4_/Ag_3_PO_4_@WO_3_-0.25 composites is about 36.225 and 20.525 emug^−1^ respectively. Such a decrease of their saturated magnetization could be attributed to the decrease in effective mass of the Fe_3_O_4_ in these cases. Fortunately, the magnetism of these hybrid nanostructures was still strong enough to be separated easily from solution with the help of an external magnetic field.

**Table 1:** Magnetic properties of Fe_3_O_4_

Parameter	Upward part	Downward part	Average	Hysteresis parameter
Mr (emu/)	0.2394	0.207125	0.223275	Remanent magnetization: M H = 0
S	0.007	0.006	0.006	Squarenses: Mr/Ms
S*	0.134	0.157	0.146	1 − (Mr/Hc)(1/slop at Hc)
Ms (emu/g)	36.225	36.2	36.225	Saturation magnetization: maximum M measure
Hc Oe	2.36	− 2.04	2.20	Coercive field: field at which M/H changes sign

**Table 2:** Magnetic properties of the Fe_3_O_4_Ag_3_PO_4_@WO_3_

Parameter	Upward part	Downward part	Average	Hysteresis parameter
Mr (emu/)	0.0096	0.0084	0.0090	Remanent magnetization: M H = 0
S	0.005	0.004	0.003	Squarenses: Mr/Ms
S*	0.118	0.136	0.127	1 − (Mr/Hc)(1/slop at Hc)
Ms (emu/g)	20.520	20.53	20.525	Saturation magnetization: maximum M measure
Hc Oe	2.00	− 1.75	1.87	Coercive field: field at which M/H changes sign

#### Fourier transforms infrared spectroscopy

**Fig. 6:**
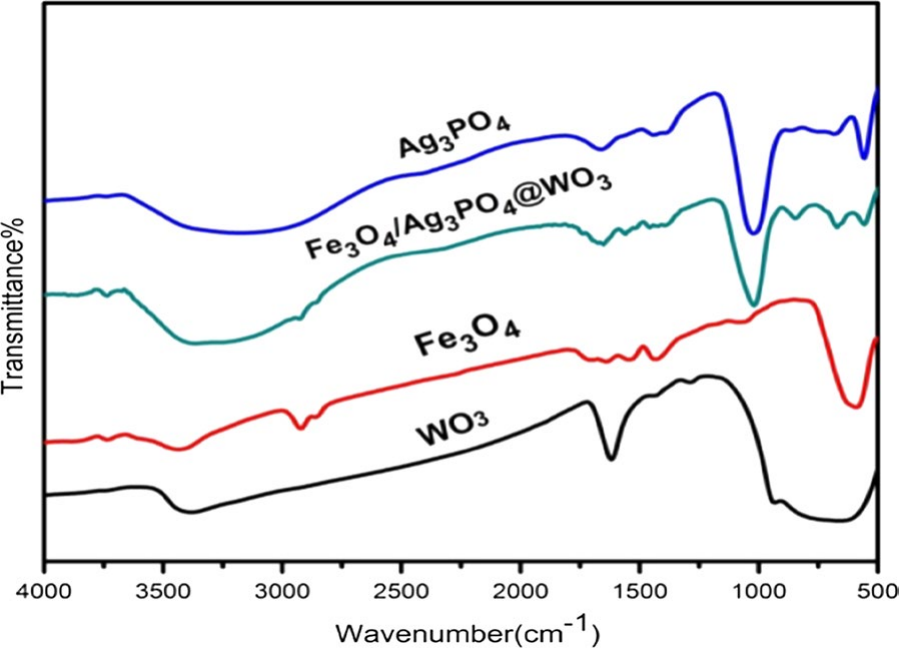
FTIR Spectrums of the WO_3_, Fe_3_O_4_, Fe_3_O_4_/Ag_3_PO_4_@WO_3_ and Ag_3_PO_4_, Composite

The typical vibration peaks of the Fe_3_O_4_, Ag_3_PO_4_, WO_3_, and Fe_3_O_4_/Ag_3_PO_4_@WO_3_ composite are shown in the FT-IR spectrum in (Fig. 6). In the spectra of the functionalized Fe_3_O_4_, the presence of OA layer can be confirmed. The bands at 2840 cm^−1^ and 2917 cm^−1^ can be ascribed to the stretching modes of –CH_2_ and –CH_3_ of the OA, respectively. The vibrations at 1699 cm^−1^ and 1454 cm^−1^ can be assigned to the stretching modes of –C=O and –C=C– of the OA, respectively. The characteristic band of the pure Fe_3_O_4_ usually appears at 570 cm^−1^, in the case of the present sample, this peak was shifted to 579 cm^−1^, due to the functionalization process. In the spectra of Ag_3_PO_4_, the intense absorption peak at 1016 cm ^−1^ is ascribed to the stretching vibration of the phosphate (PO_4_^3−^) group, and the absorption peaks at 574 and 538 cm^−1^ is ascribed to the bending vibration of the phosphate (PO_4_^3−^) group. The absorption peaks at 3450 and 1660 cm^−1^ are corresponding to the ^−^OH stretching and bending vibrations of physically absorbed H_2_O molecules, respectively. The precursor of WO_3_ has strong bands in the 500–900 cm^−1^ region are assigned to the ν(O–W–O) stretching mode [37]. A band at 950 cm^−1^ was observed in the spectra of the terminal of vibrations W=O groups that were changed from the W–O bond on the surface of WO_3_ or in the grain boundaries in WO_3_ [38, 39]. Bands at 3380 and 1618 cm^−1^ can be indexed to the ^−^OH stretching σ(O–H) bending vibrations of coordinated water [37]. In the spectra of Fe_3_O_4_/Ag_3_PO_4_@WO_3_ composite, all the characteristic peaks of Fe_3_O_4_, Ag_3_PO_4_ and WO_3_ are present with a red shift for the peak of Fe_3_O_4_ (from 579 to 675 cm^−1^) and blue shift for the peaks of Ag_3_PO_4_ (from 1028 to 1013 cm^−1^). The FT-IR analysis further confirms the fabrication of the Fe_3_O_4_/Ag_3_PO_4_@WO_3_ composite.

#### UV–vis diffuse reflectance spectroscopy and band structure

**Fig. 7:**
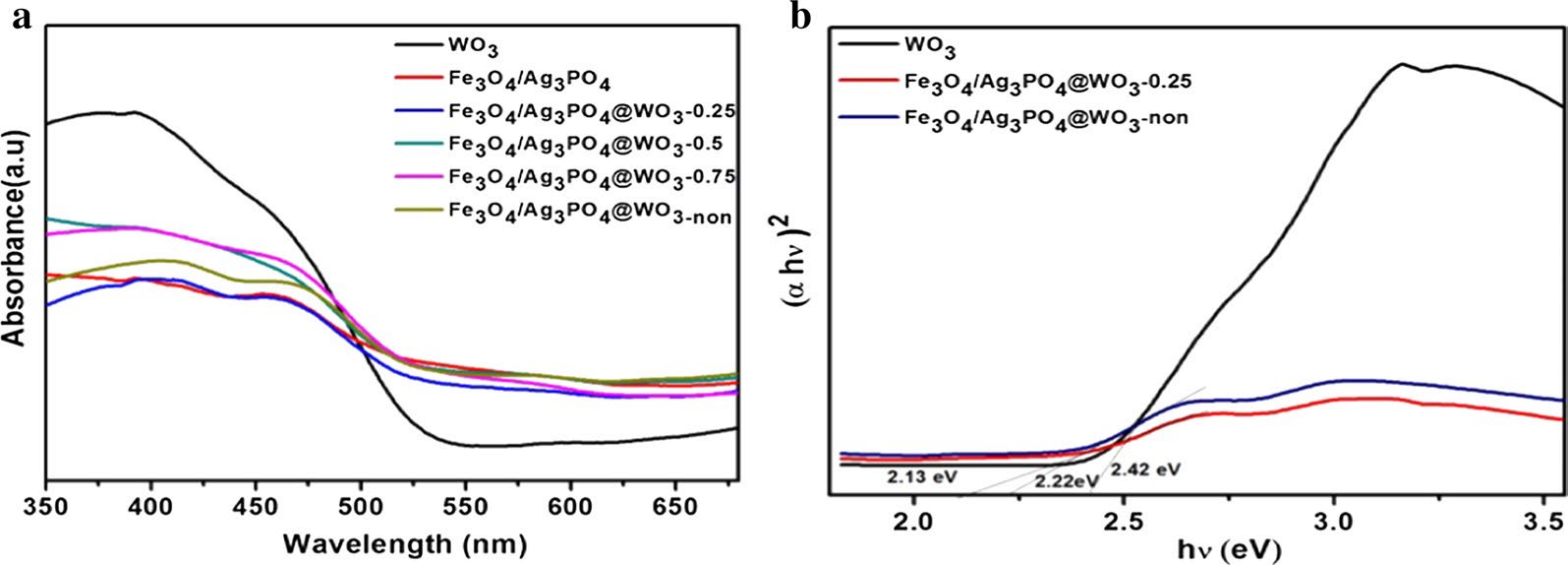
**a** UV–visible absorption spectra WO_3_, Fe_3_O_4_/Ag_3_PO_4_@WO_3_-0.25 mg, Fe_3_O_4_/Ag_3_PO_4_@WO_3_-0.50 and Fe_3_O_4_/Ag_3_PO_4_@WO_3_-0.75 mg and Fe_3_O_4_/Ag_3_PO_4_@WO_3_-0.25 mg-non and **b** plots of (αhv)^2^ versus energy (hv) for the band gap energy of WO3, Fe3O4/Ag3PO4@WO3 and Fe3O4/Ag3PO4@WO3-non

The study of the light absorption behaviors clearly shows the efficient photocatalytic properties of the semiconductor [40, 41]. The light absorption behaviors of the synthesized photocatalysts were studied by DRS analysis, and the results are shown in (Fig. 7a). The results show that, the light absorptivity of the products shown a trend to increase in the range of 200–520 nm and reached a peak at 510 nm, which belonged to the visible region. The band gap of the synthesized composites was determined by a plot of (αhv) ^2^ versus energy (hv), (Fig. 7b). The band gaps were estimated to be 2.42 eV, 2.22 eV and 2.13 eV for WO_3_, Fe_3_O_4_/Ag_3_PO_4_@WO_3_ and Fe_3_O_4_/Ag_3_PO_4_@WO_3_-non, respectively. The as synthesized Fe_3_O_4_/Ag_3_PO_4_@WO_3_ composite exhibited the narrower band gap, which is beneficial to its enhanced photocatalytic activity.

#### Evaluation of antibacterial activity

**Fig. 8:**
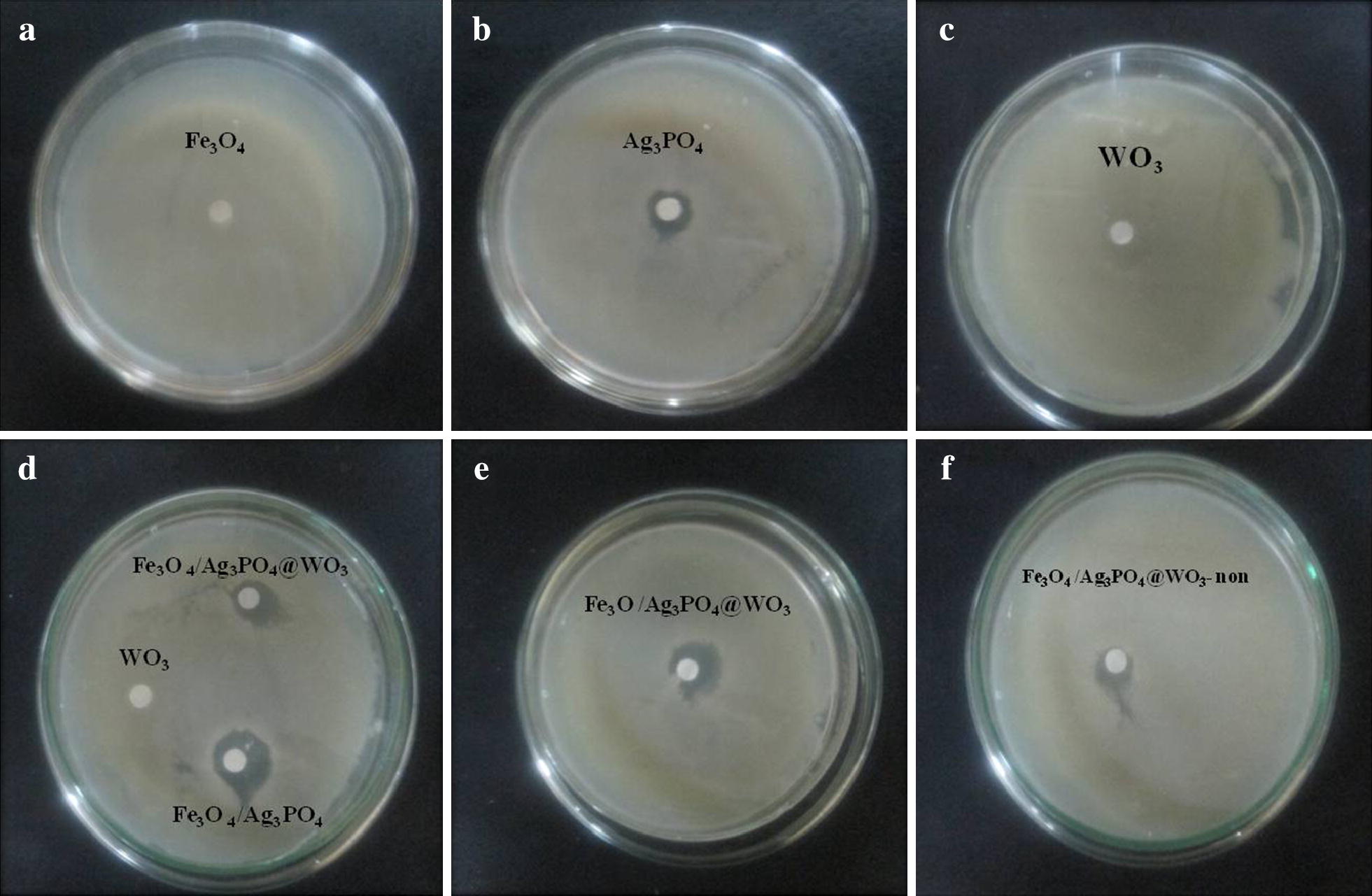
**a**–**f** Images of agar plates containing *S. aureus* after treated by the synthesized composites

Concerning the prospective application of Fe_3_O_4_/Ag_3_PO_4_/WO_3_, the antibacterial properties of the as-synthesized catalyst were investigated in the present work. *S. aureus* even was chosen as representative microorganism. Following the findings above, it was presumed that successful deposition of Ag_3_PO_4_ imparted antibacterial functions on the nanocomposites [42–44]. To further assess this function, we employed the test method of antimicrobial in term of the inhibitory zone. In a control sample, bacterial colony growth was induced on an Agar dish as illustrated in (Fig. 8a–f), following interfacial contact between the test samples and the Agar plate, which should lead to inhibition of the bacterial growth. The radius of the inhibition zones was measured. As seen in Fig. 8a and c, no bacterial growth inhibition observed in the presence of both Fe_3_O_4_ and WO_3_. The inhibition zone radius for the synthesized composites are ∼ 13, 19 and 17 mm for Ag_3_PO_4_, Fe_3_O_4_/Ag_3_PO_4_, and Fe_3_O_4_/Ag_3_PO_4_@WO_3_, respectively. Showed that all Ag_3_PO_4_-based composite has significant bactericidal activity against *S. aureus.* The comparative between Fe_3_O_4_/Ag_3_PO_4_@WO_3_ and Fe_3_O_4_/Ag_3_PO_4_@WO_3_-non composite shows inhibition zone of 12 mm and 17 mm, respectively.

#### Study of photocatalytic activity

**Fig. 9:**
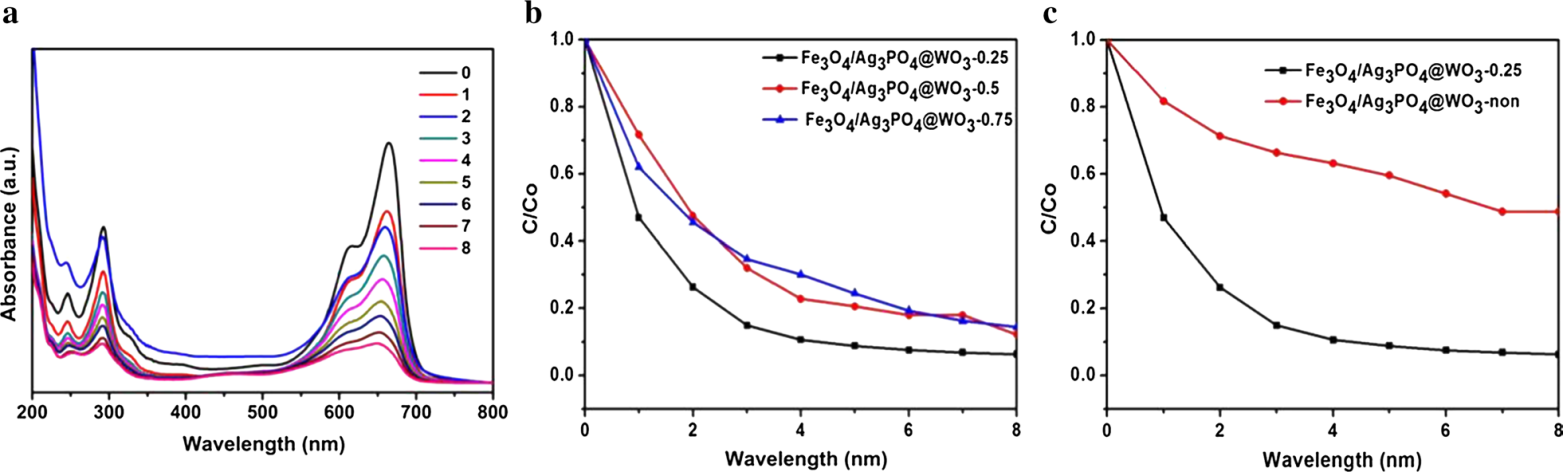
**a** Changes of MB in aqueous Solution of Fe_3_O_4_/Ag_3_PO_4_@WO_3_, **b** Comparison of photocatalytic activity of synthesized photocatalysts for degradation of MB under simulated sunlight irradiation, **c** Comparison between Fe_3_O_4_/Ag_3_PO_4_ @WO_3_ and Fe_3_O_4_/Ag_3_PO_4_ @WO_3_-non

For the evaluation of the photocatalytic activity of the synthesized composites, MB was used as a representative sample. Figure 9a shows the photocatalytic activities of the Fe_3_O_4_/Ag_3_PO_4_@WO_3_ photocatalysts with different ratios of Ag_3_PO_4_ under simulated light irradiation and the MB solution shows light absorption edge at λ_max_ of 664 nm. In order to investigate the optimum amount of Ag_3_PO_4_ on the synthesized composite, Fe_3_O_4_/Ag_3_PO_4_@WO_3_ with different Ag_3_PO_4_ was synthesized, and their photocatalytic activity against MB degradation was studied, and the results are shown in (Fig. 9b). As seen Fe_3_O_4_/Ag_3_PO_4_@WO_3_-0.25 composite, exhibited the higher photocatalytic activity compared with Fe_3_O_4_/Ag_3_PO_4_@WO_3_-0.50 and Fe_3_O_4_/Ag_3_PO_4_@WO_3_-0.75. This is due to the best charge separation and transforming of Fe_3_O_4_/Ag_3_PO_4_@WO_3_-0.25.

To get deeply in the study of the photocatalytic efficiency of Fe_3_O_4_/Ag_3_PO_4_@WO_3_-0.25, its catalytic performance was compared with the Fe_3_O_4_/Ag_3_PO_4_@WO_3_-non composite. The results are shown in Fig. 9c. As clearly been seen Fe_3_O_4_/Ag_3_PO_4_@WO_3_-0.25 displayed higher photocatalytic activity. 90% of MB degraded within 6 min, while the non-functionalized composite required 8 min to degrade 50% of the dye. The results of the MB degradation revealed that, the Fe_3_O_4_/Ag_3_PO_4_@WO_3_-0.25 composite was exhibited the highest photocatalytic performance. This is because; the functionalization process significantly induced the electron transfer properties of the Fe_3_O_4_. Therefore, enhanced the electron–hole separation and transfer in the Fe_3_O_4_/Ag_3_PO_4_@WO_3_ composite.

#### Electrochemical impedance spectroscopy (EIS)

**Fig. 10:**
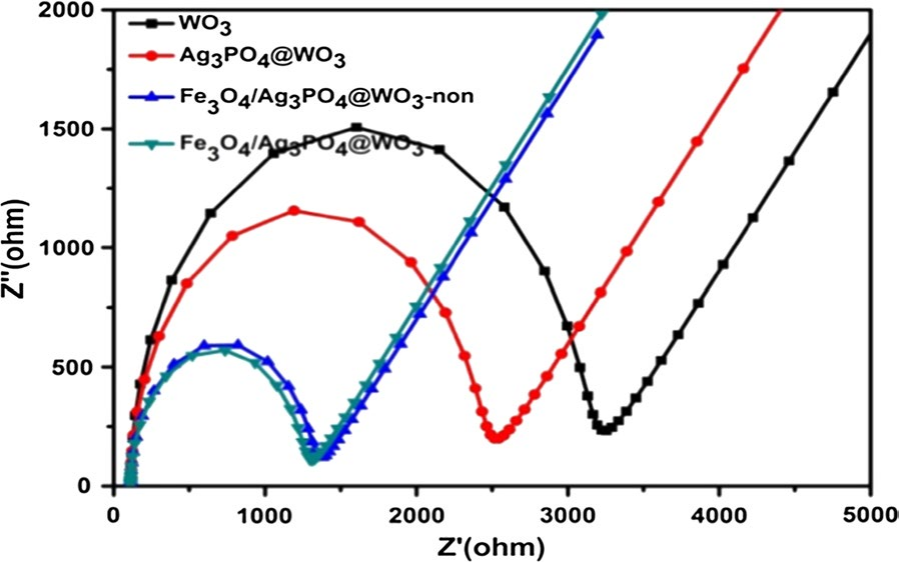
The electrochemical impedance spectroscopy of WO_3_, Ag_3_PO_4_@WO_3_, Fe_3_O_4_/Ag_3_PO_4_@WO_3_-non and Fe_3_O_4_/Ag_3_PO_4_@WO_3_

The electrochemical impedance spectroscopy of the WO_3_, Ag_3_PO_4_@WO_3_, Fe_3_O_4_/Ag_3_PO_4_@WO_3_-non and Fe_3_O_4_/Ag_3_PO_4_@WO_3_ composite was carried out to further investigated the charge transfer and recombination processes in the Fe_3_O_4_/Ag_3_PO_4_@WO_3_ composites under simulated light irradiation, and the result shown in (Fig. 10), a smaller arc radius can be observed on the EIS Nyquist plot of Fe_3_O_4_/Ag_3_PO_4_@WO_3_-non and Fe_3_O_4_/Ag_3_PO_4_@WO_3_ composite compared with WO_3_ and Ag_3_PO_4_@WO_3_ indicating that a more effective separation of the photogenerated electron/hole pairs and a faster interfacial charge transfer occurred on the surface of the Fe_3_O_4_/Ag_3_PO_4_@WO_3_ composite.

#### Kinetic study

**Fig. 11:**
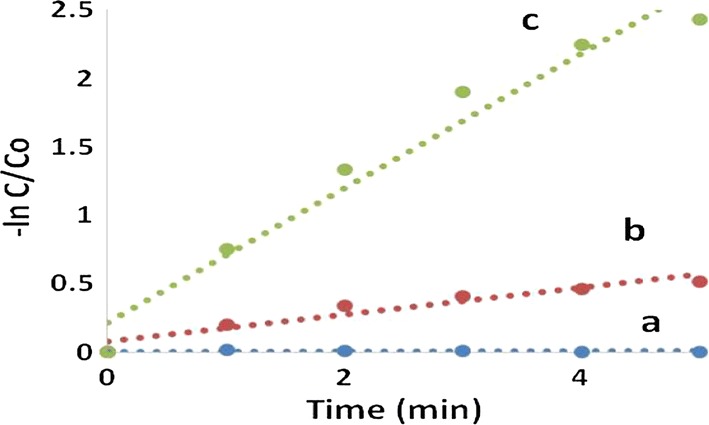
The kinetic fit for the degradation of MB with **a** WO_3_, **b** Fe_3_O_4_/Ag_3_PO_4_@WO_3_-non and **c** Fe_3_O_4_/Ag_3_PO_4_@WO_3_

In order to investigate the kinetic behavior of the synthesized photocatalysts on the degradation of MB under simulated sunlight irradiation, ln (C/Co) for MB is plotted versus irradiation time according to the following first-order kinetic model 2:

2 $${ \ln }\left( {{\text{C}}/{\text{Co}}} \right) = - {\text{kt}}$$

where k is the degradation rate constant, Co and C the initial concentration and the concentration at different irradiation time t of the organic dye respectively. From the result presented in (Fig. 11), the disappearance of MB over the Fe_3_O_4_/Ag_3_PO_4_@WO_3_ synthesized photocatalysts under simulated light irradiation is shown to fit a pseudo first order kinetics pattern, with degradation rate constants of 0.0018, 0.098 and 0.4906 min^−1^ and R^2^: 0.704, 0.918 and 0.959 for WO_3_, Fe_3_O_4_/Ag_3_PO_4_@WO_3_-non and Fe_3_O_4_/Ag_3_PO_4_@WO_3_ respectively. It is concluded that the degradation of MB over the as-synthesized Fe_3_O_4_/Ag_3_PO_4_@WO_3_ under light irradiation is five times faster than that of Fe_3_O_4_/Ag_3_PO_4_@WO_3_-non, indicating the enhanced charge separation in the Fe_3_O_4_/Ag_3_PO_4_@WO_3_ composite compared with Fe_3_O_4_/Ag_3_PO_4_@WO_3_-non.

#### Stability study

**Fig. 12:**
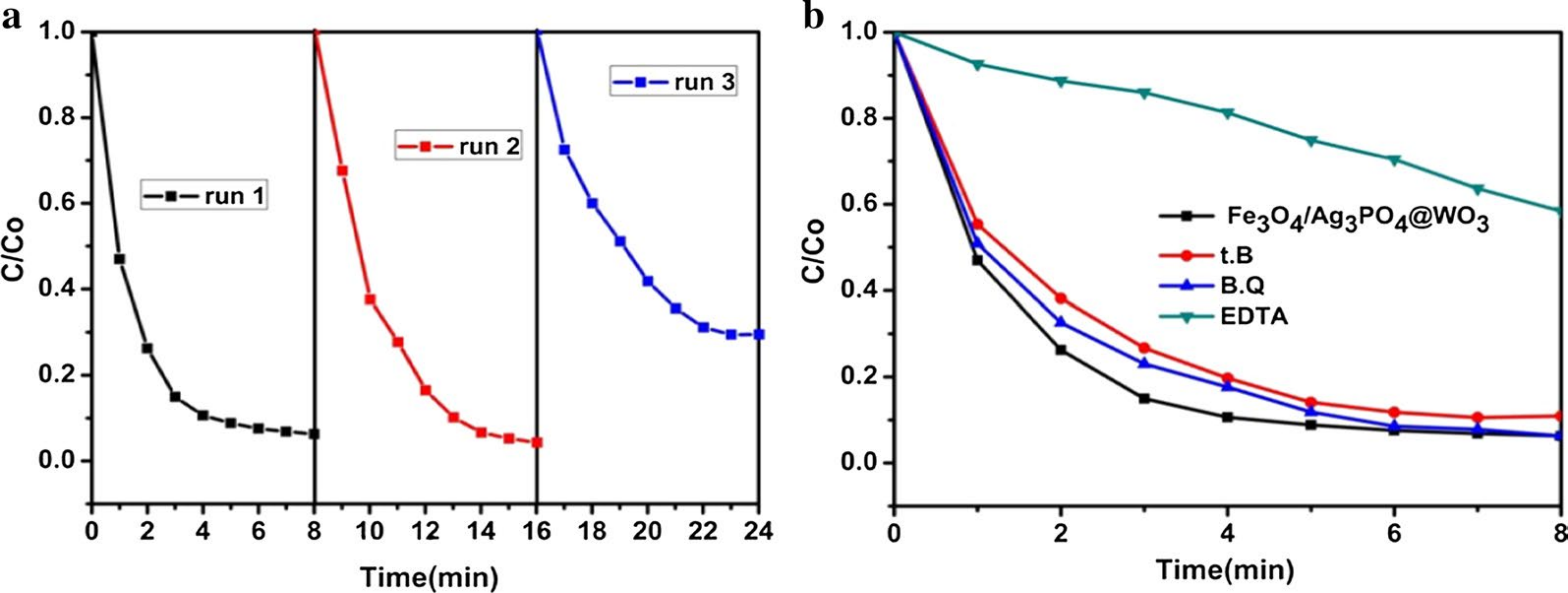
**a** Stability study on the photocatalytic degradation of MB solution over Fe_3_O_4_/Ag_3_PO_4_@WO_3_-0.25 nanocomposite under simulated sunlight irradiation, **b** the degradation of MB in the presence of different radical scavengers with Fe_3_O_4_/Ag_3_PO_4_ @WO_3_ composite

The stability of a photocatalyst is important for its practical application. The stability and recyclability of Fe_3_O_4_/Ag_3_PO_4_@WO_3_ are evaluated by additional experiments to degrade MB under simulated light irradiation cycled for three times. Figure 12a, shows the repeated visible light photocatalytic activity of Fe_3_O_4_/Ag_3_PO_4_@WO_3_, it can be observed that composite have good stability for the degradation of MB under simulated light irradiation during three cycles.

#### Active species responsible for MB degradation

The recombination of photogenerated electron–hole pairs is an important limiting factor for the performance of photocatalysts [45, 46]. To study the photocatalytic mechanism of the Fe_3_O_4_/Ag_3_PO_4_/WO_30_-0.25 composite in detail, the effects of different scavengers on the decomposition of dye molecules were investigated. In order to determined the predominant reactive oxygen species in the photocatalytic process. In this study, *p*-benzoquinone (BZQ), disodium ethylenediamine tetraacetate (Na_2_-EDTA) and tert-butanol (t.B) were used as scavengers for O_2_^·−^, h^+^, and ^·^OH, respectively [47]. As seen in (Fig. 12b), the addition of B.Q and t.B has slight effect on the inhibition of the dye degradation. Introducing EDTA to the reaction system the photocatalytic activity significantly reduced. Which indicate that the photocatalytic reaction mainly depends on the h^+^. While O_2_^·−^ and ^·^OH have negligible effect. Herein, the highly enhanced photodegradation and antimicrobial activities achieved on Fe_3_O_4_/Ag_3_PO_4_@WO_3_ can be assigned to its composite structure.

**Fig. 13:**
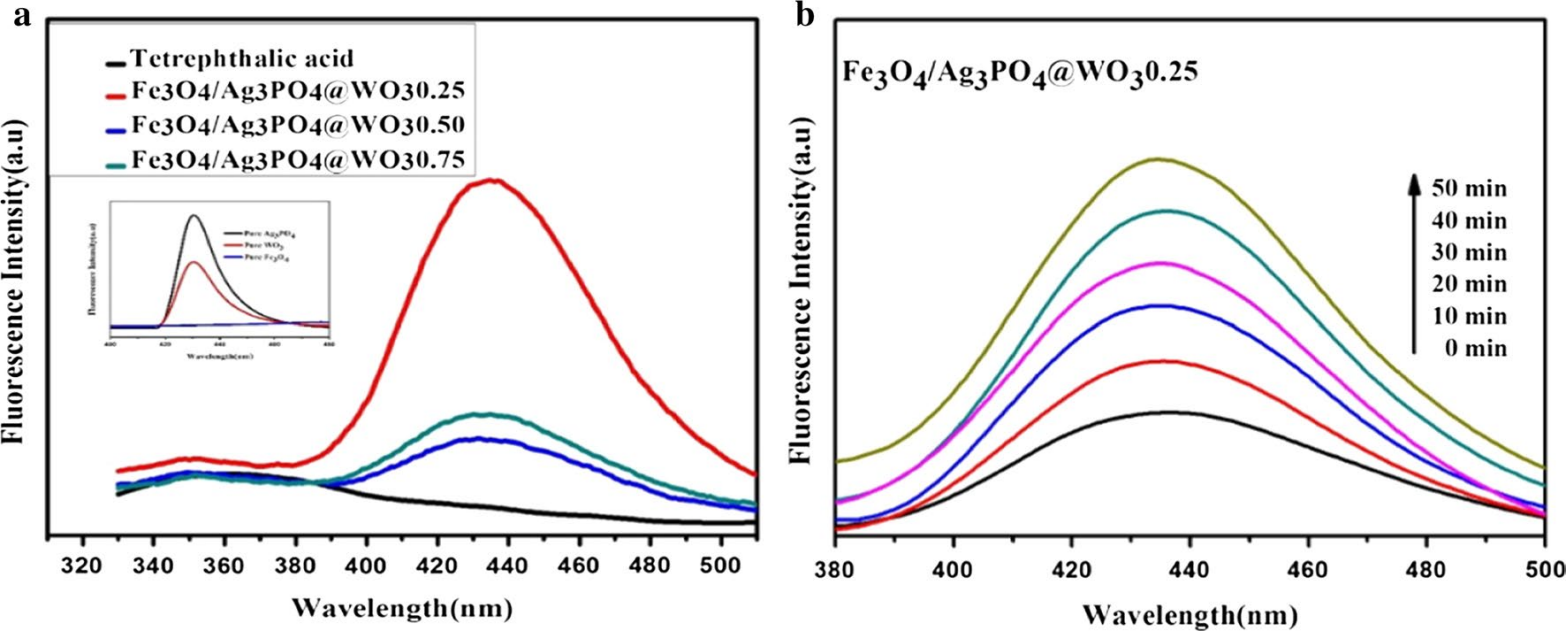
**a** PL spectra of the aqueous basic solution of terephthalic acid and Fe_3_O_4_/Ag_3_PO_4_@WO_3_ (0.25, 0.50 and 0.75) with an excitation at 365 nm under different composites for 50 min. Inset of **a** is the PL spectra of pure sample Ag_3_PO_4_, WO_3_ and Fe_3_O_4_ and **b** PL spectra of changing with irradiation time for the case of the Fe_3_O_4_/Ag_3_PO_4_@WO_3_-0.25

The formation of ^·^OH on the surface photo-illuminated of composite Fe_3_O_4_/Ag_3_PO_4_@WO_3_ (0.25, 0.50 and 0.75) was further confirmed by the PL technique using terephthalic acid as a probe molecule. Figure 13a shows that an obvious difference in PL intensity at about ∼ 425-430 nm was observed using different catalysts. It was clear that the formation rate of ^·^OH on the Fe_3_O_4_/Ag_3_PO_4_@WO_3_-0.25 was higher than that of other composite. This implied that the former has higher photocatalytic activity than the latter. Moreover, the inset of (Fig. 13a) the pure Ag_3_PO_4_ exhibited higher PL intensity than pure WO_3_ and pure Fe_3_O_4_, suggesting that doping of Ag_3_PO_4_ with WO_3_ and Fe_3_O_4_ was a good route to accelerate the interfacial charge transfer and inhibit the recombination of electron–hole pairs, which resulted in the increase in ^·^OH formation. Moreover, the Fig. 13b depicts the change of PL spectra with irradiation time for the case of Fe_3_O_4_/Ag_3_PO_4_@WO_3_-0.25. A gradual increase in PL intensity was observed with increasing irradiation time, which suggested that the fluorescence was caused by chemical reactions of terephthalic acid with ^·^OH formed during photo-illuminated reactions. Thus, these results confirmed the evidence of ^·^OH formation and indeed participated in degradation process.

#### Probable mechanism

**Fig. 14:**
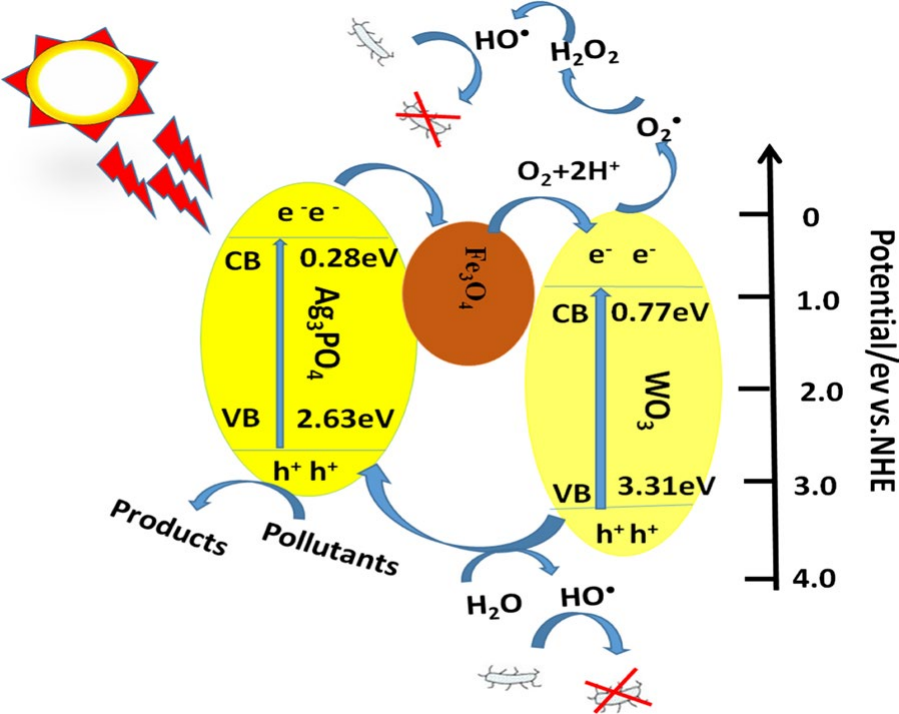
Scheme diagrams for the enhanced activity of Fe_3_O_4_/Ag_3_PO_4_@WO_3_-0.25 photocatalysts

As illustrated in Fig. 14 the semiconductors of WO_3_ (with an optical band gap of 2.44 eV and Ag_3_PO_4_ (with an optical band gap of 2.35 eV, especially, the redox potentials (CB and VB) of WO_3_ are more positive than those of Ag_3_PO_4_. The electrons from the CB of Ag_3_PO_4_ can migrate to that of WO_3_ and the photogenerated holes could migrate from the VB of WO_3_ to that of Ag_3_PO_4_. The holes could directly oxidize the organic dyes adsorbed on Ag_3_PO_4_ surface [48–50] and the electrons could be consumed through a multi-electron reaction with oxygen (O_2_ + 2H^+^ + 2e^−^ → H_2_O_2_, E^0^ = + 0.682 V vs. NHE) [51]. The produced H_2_O_2_ reacts with an additional electron to produce^·^OH (H_2_O_2_ + e^−^ → OH^−^ + ^·^OH) [52], which could accelerate the antimicrobial activity.

The major reaction steps in this Fe_3_O_4_/Ag_3_PO_4_@WO_3_ composite photocatalytic mechanism under visible light irradiation are summarized by Eqs. 3–9.

3 $${\text{WO}}_{ 3} + {\text{h}}\upnu \to {\text{WO}}_{ 3} *$$

4 $${\text{WO}}_{ 3} * + {\text{Ag}}_{ 3} {\text{PO}}_{ 4} \to {\text{WO}}_{ 3}^{ + \cdot } + {\text{Ag}}_{ 3} {\text{PO}}_{ 4} \left( {\text{e}} \right)$$

5 $${\text{Ag}}_{ 3} {\text{PO}}_{ 4} \left( {\text{e}} \right) + {\text{O}}_{ 2} \to {\text{Ag}}_{ 3} {\text{PO}}_{ 4} + {\text{O}}_{ 2}^{ - \cdot }$$

6 $${\text{O}}_{ 2}^{ - \cdot } + {\text{H}} + \to^{ \cdot } {\text{OOH}}$$

7 $$^{ \cdot } {\text{OOH}} + {\text{Ag}}_{ 3} {\text{PO}}_{ 4} \left( {\text{e}} \right) + {\text{H}}^{ + } \to {\text{H}}_{ 2} {\text{O}}_{ 2} + {\text{Ag}}_{ 3} {\text{PO}}_{ 4}$$

8 $${\text{H}}_{ 2} {\text{O}}_{ 2} + {\text{Ag}}_{ 3} {\text{PO}}_{ 4} \left( {\text{e}} \right) \to^{ \cdot } {\text{OH}} + {\text{OH}}^{ - } + {\text{Ag}}_{ 3} {\text{PO}}_{ 4}$$

9 $${\text{Organic pullutant with}}^{ \cdot } {\text{OH}},{\text{ O}}_{ 2}^{ - \cdot } {\text{or H}}_{ 2} {\text{O}}_{ 2} \to \to$$

degraded or mineralized products and bacterial activity.

At the same time, the enhanced photocatalytic activity could be expected the composites Fe_3_O_4_/Ag_3_PO_4_/WO_3_-0.25 catalyst due to the effective separation of photogenerated electron–hole pairs. According to the plot of (Ahν)^2^ vs. hν, the band gaps (Eg) of Ag_3_PO_4_ and WO_3_ are estimated to be 2.35 and 2.44 eV, respectively. The band structure of Fe_3_O_4_/Ag_3_PO_4_/WO_3_ composites can be estimated according to the empirical equations as shown below:

10 $${\text{VB}} = {\text{X}}{-}{\text{E}}^{\text{e}} + 0. 5 {\text{ E}}_{\text{g}}$$

11 $${\text{CB}} = {\text{VB}} - {\text{E}}_{\text{g}}$$

where EVB and ECB are the valence and conduction band edge potentials, respectively; χ is the electronegativity of the semiconductor; Ee is the energy of free electrons on the hydrogen scale (about 4.5 eV vs. NHE). The χ values for bare Fe_3_O_4_, Ag_3_PO_4_ and WO_3_ are 5.78, 5.96 and 6.49 eV and, respectively [53–55]. Thus, the EVB of Fe_3_O_4_, Ag_3_PO_4_ and WO_3_ have been calculated to be 2.18, 2.63 and 3.31 eV vs. NHE, and the corresponding ECB are 0.38, 0.28 and 0.77 eV vs. NHE, respectively.

## Conclusions

In this work, novel Fe_3_O_4_/Ag_3_PO_4_@WO_3_ photocatalysts were successfully synthesized via in situ ion-exchange method, and employed in the simulated light degradation of organic contaminants (Methylene Blue). The as synthesized composite exhibited enhanced photocatalytic activity compared with Fe_3_O_4_/Ag_3_PO_4_@WO_3_-non and the bare WO_3_. In addition, the effect of the Ag_3_PO_4_ amount on the photocatalytic activity of the Fe_3_O_4_/Ag_3_PO_4_@WO_3_ was investigated. The Fe_3_O_4_/Ag_3_PO_4_@WO_3_-0.25 exhibited the higher photocatalytic activity. The antibacterial behaviors of the synthesized composite were studied. All the Ag_3_PO_4_, Fe_3_O_4_/Ag_3_PO_4_ and Fe_3_O_4_/Ag_3_PO_4_@WO_3_ based composites exhibited enhancement in bactericidal efficiency. The oleic acid functionalized composite Fe_3_O_4_/Ag_3_PO_4_@WO_3_ also exhibited the high inhibition zone. The reactive species trapping study revealed that, the hole played the main role on the photocatalytic activity enhancement.
